# Whole-body volatile organic compound profiling reveals metabolic pathway perturbations in a murine polymicrobial sepsis model: a feasibility study

**DOI:** 10.3389/fmed.2026.1978491

**Published:** 2026-10-14

**Authors:** Wan-yi Feng, Yu Chen, Xiu-ya Zhang, Rong Fu, Qin Hu, Feng-feng Mo, Jia-feng Wang

**Affiliations:** 1 Department of Anesthesiology, Changhai Hospital, Naval Medical University, Shanghai, China; 2 Department of Naval Nutrition and Food Hygiene, Faculty of Naval Medicine, Naval Medical University, Shanghai, China; 3 Key Laboratory of Biological Defense, Ministry of Education, Naval Medical University, Shanghai, China

**Keywords:** cecal ligation and puncture, gas chromatography-mass spectrometry, gut microbiome, immunometabolism, lipid peroxidation, sepsis, volatile organic compounds, whole-body headspace volatilomics

## Abstract

Sepsis rewires host metabolism, and these changes may be readable in the volatile organic compounds (VOCs) released from the body. Whether whole-body VOC profiles can be mapped onto specific disrupted biochemical pathways has not been tested. In this discovery-phase, single-time-point feasibility study, polymicrobial sepsis was induced by cecal ligation and puncture (CLP) in C57BL/6 mice (14 CLP, 10 sham controls). Whole-body headspace gases were collected 12 h after surgery and analysed by thermal desorption-gas chromatography-mass spectrometry. After preprocessing, 113 features were tested univariately with Benjamini-Hochberg correction and by orthogonal partial least squares discriminant analysis (OPLS-DA). Candidate VOCs (FDR < 0.1, |log2FC| > 0.26, VIP > 1.0) were annotated against PubChem, KEGG COMPOUND, and the Human Metabolome Database. OPLS-DA separated the groups (R2X = 0.489, R2Y = 0.981, Q2 = 0.777; *P* = 0.001 by 1,000 permutations), and leave-one-out cross-validation classified 95.8% of animals correctly, though *n* = 24. Of 54 candidate VOCs, 18 had established endogenous origins; 16 fell and 2 rose in septic mice. Pathway mapping indicated four perturbed domains: lipid peroxidation (12 depleted VOCs), ketone body metabolism (3 decreased), cholesterol/isoprenoid metabolism (isoprene, decreased), and putative gut microbiota-associated metabolism (toluene and carbon disulfide, increased). All 18 endogenous VOCs kept candidate status after environment-derived VOCs were removed. Because whole-body headspace volatiles superimpose tissue metabolism, ventilation, activity, and background, these results suggest the septic volatilome may be mapped onto distinct pathway perturbations; ventilation and activity effects cannot be excluded. These findings generate testable hypotheses for non-invasive metabolic phenotyping in sepsis.

## 1 Introduction

Sepsis, defined as life-threatening organ dysfunction caused by a dysregulated host response to infection, kills an estimated 11 million people each year (1, 2). Diagnosis still depends on blood cultures that take 24–48 h and on nonspecific clinical criteria such as the Sequential Organ Failure Assessment (SOFA) score. The delay creates a practical need for rapid, non-invasive tools that can track the metabolic status of critically ill patients at the bedside (3).

Exhaled breath and cutaneous emanations contain thousands of volatile organic compounds (VOCs) that originate from systemic metabolic processes and reach the lungs or skin through the bloodstream (4, 5). The composition of this volatilome shifts in lung cancer, chronic obstructive pulmonary disease, liver cirrhosis, and systemic inflammation (6–8). VOCs are terminal end-products of biochemical reactions, so their profiles report on the state of upstream metabolic pathways. This makes volatilomics attractive for non-invasive metabolic phenotyping.

Two earlier studies used ion mobility spectrometry (IMS) to profile breath VOCs in rodents. Fink et al. applied multicapillary column IMS in rats after cecal ligation and incision and identified discriminatory VOCs such as 3-pentanone, acetone, and 2-hexanone across sepsis, endotoxemia, and hemorrhagic shock models (9). Guaman et al. combined IMS with solid-phase microextraction GC-MS in a lipopolysaccharide-induced rat endotoxemia model and reported 15 discriminatory compounds with a bootstrap-validated classification accuracy of 85% (10). Both studies showed that breath VOCs can separate septic from control animals, but neither traced the identified compounds back to their pathophysiological origins.

GC-MS offers higher chemical resolution than IMS, which supports more definitive structural annotation and, in turn, pathway-level interpretation (11). Combined with multivariate statistics and metabolite-centric pathway enrichment, an approach common in liquid-phase metabolomics but rarely applied to volatilomics (12–14), GC-MS can connect empirical biomarker discovery to underlying biology. Sepsis involves deep immunometabolic reprogramming, and the affected processes include fatty acid oxidation, ketogenesis, and the production of microbiome-associated metabolites (1, 2). We therefore used thermal desorption GC-MS (TD-GC-MS) to profile systemic micro-environmental VOCs in a well-characterised murine cecal ligation and puncture (CLP) model of polymicrobial sepsis. Our primary aim was to test whether whole-body VOC profiling identifies specific metabolic pathways perturbed during acute sepsis. Two boundaries follow from the sampling design. First, whole-body headspace VOCs are composite signals from superimposed sources, including host tissue metabolism, ventilation and physical activity, the gut microbiota, and ambient background; their abundance cannot be equated directly with metabolic pathway activity inside tissues. Second, the setup cannot attribute any compound to a specific organ. The study is therefore designed to generate hypotheses rather than to confirm *in vivo* pathway perturbations.

## 2 Materials and methods

### 2.1 Animals and experimental design

All experimental protocols were approved by the institutional Animal Ethics Committee (Approval No. LL20230715; July 13, 2023) and followed national guidelines for the care and use of laboratory animals. Healthy male C57BL/6 mice (6–8 weeks old, 20–25 g) were housed under specific pathogen-free conditions (22 ± 2 °C, 50%–60% humidity, 12-h light/dark cycle) with free access to a standard rodent diet and water. After 1 week of acclimatisation, mice were randomly allocated to a polymicrobial sepsis cohort induced by CLP (*n* = 14) or a sham-operated control cohort (*n* = 10).

### 2.2 Cecal ligation and puncture

Anesthesia was induced with 4% isoflurane in 100% oxygen (1.0 L/min) and maintained at 1.5%–2.0% (0.5 L/min). After abdominal shaving and disinfection, a 1.5-cm midline laparotomy was performed. The cecum was exteriorised, ligated at its distal half with 4-0 silk suture, and punctured through-and-through with an 18-gauge needle. A small droplet of fecal content was extruded through the puncture sites to ensure reproducible peritoneal contamination. The cecum was returned to the abdominal cavity and the wall was closed in layers. Ropivacaine was infiltrated along the incision for analgesia, and 1 mL of pre-warmed sterile saline was injected subcutaneously for fluid resuscitation. Sham mice underwent the same laparotomy and bowel manipulation without ligation or puncture. No perioperative antibiotics were given, so the model represents severe polymicrobial sepsis without antibiotic rescue.

### 2.3 Systemic micro-environmental VOC sampling

At 12 h after surgery (acute-phase sepsis), mice were placed individually into hermetically sealed glass chambers (2 L) with low volatile emission. The chambers were continuously purged with high-purity air (200 mL/min). After 30 min of equilibration, whole-body headspace gases were collected onto Tenax TA thermal desorption tubes (Markes International, Llantrisant, UK) at 100 mL/min for 20 min. Ambient air blanks from empty chambers were collected with every batch of biological samples, using an identical chamber and flow protocol, to monitor background and guide classification of exogenous compounds. They were used qualitatively, to flag compounds present in ambient air, and not for quantitative subtraction. Sampling order was randomised with respect to group, so the two cohorts were not processed in separate blocks. Tubes were capped immediately, sealed, and stored at 4 °C until analysis within 24 h.

### 2.4 TD-GC-MS analysis

Volatile analysis used a TD-100 thermal desorption unit (Markes International) coupled to a 7890B gas chromatograph and a 5977B mass spectrometer (Agilent Technologies, Santa Clara, CA, USA). Thermal desorption was performed at 280 °C for 10 min at a trap flow of 50 mL/min. Separation used a DB-624 capillary column (60 m × 0.32 mm i.d. × 1.8 µm film thickness) with helium at a constant flow of 1.0 mL/min. The oven was held at 35 °C for 5 min, ramped at 5 °C/min to 230 °C, and held for 10 min. Mass spectra were acquired in full scan mode (m/z 35–350) with electron ionization at 70 eV. Compounds were identified by matching spectra against the NIST 14 library with a minimum match factor of 800. Technical replicates were not performed: thermal desorption is a one-shot technique in which the sorbent tube is exhausted by a single desorption (32), as is standard practice in breath volatilomics. Precision was instead monitored with pooled quality-control samples, ambient-air blanks and retention-time stability, giving relative standard deviations below 10%. Samples were injected in randomized order, and a pooled quality-control sample (equal aliquots of all 24 samples) was run every 10 injections to monitor retention-time drift and response stability. An empty tube was run at the end of each batch to assess carryover; no carryover above the blank level was detected. Retention-time alignment (tolerance 0.1 min) and peak picking (peak width 5–30 s) used MetaboAnalyst 6.0 defaults. In line with Metabolomics Standards Initiative (MSI) guidelines (28), all VOCs are reported as MSI Level 2 (putative annotation), since Level 1 confirmation requires authentic reference standards run under identical conditions.

### 2.5 Data preprocessing and statistical analysis

Raw chromatographic data were integrated automatically and then cross-checked manually. The peak-area matrix was processed in MetaboAnalyst 6.0 (14). Features with more than 50% missing values in either group were excluded, and the remaining missing values were imputed with the minimum-value fraction (min/5) method. The matrix was sum-normalised, log2-transformed, and Pareto-scaled (15). Group comparisons used two-tailed Student's t-tests or Wilcoxon rank-sum tests, chosen by Shapiro–Wilk normality testing, with Benjamini-Hochberg false discovery rate (FDR) correction. OPLS-DA was run with the ropls package (version 1.44.0, Bioconductor) in R (version 4.6.1) (15), using one predictive and one orthogonal component. Model significance was assessed by permuting the group labels 1,000 times; the number of permuted datasets reaching or exceeding the observed R2Y and Q2 was counted, and the empirical *P* value computed as (1 + exceedances)/(1 + permutations). Classification performance was assessed separately by leave-one-out cross-validation, refitting the model 24 times on 23 animals and classifying the withheld animal from its predicted response at a threshold of 0.5; accuracy, sensitivity, specificity, and area under the receiver operating characteristic curve (AUC) were reported. Candidates were defined as FDR < 0.1, |log2FC| > 0.26, and VIP > 1.0. Each candidate was classified by literature curation against PubChem (16), KEGG COMPOUND, and the Human Metabolome Database (HMDB) as endogenous (verified mammalian or microbial origin), endogenous-unknown, or exogenous. Pathway enrichment was restricted to endogenous VOCs with established biosynthetic pathways, using KEGG COMPOUND identifier mapping. Hierarchical clustering used Ward's minimum variance method with Euclidean distances. Given the modest sample size, we report the results as discovery-phase findings without a formal power calculation.

### 2.6 Sensitivity analyses

Two pre-specified sensitivity analyses tested whether the pathway-level conclusions depended on compounds whose origin could be environmental rather than biological; neither altered any other analysis step. In sensitivity analysis 1, the 20 VOCs assigned an exogenous origin by literature curation, mainly halogenated hydrocarbons and industrial solvents that also occur in ambient air and were detected in chamber blanks, were removed from the full matrix, and univariate testing with Benjamini-Hochberg correction, OPLS-DA with 1,000 permutations, and pathway enrichment were repeated on the 93 remaining features. The candidate status of each endogenous VOC was then re-evaluated against the same thresholds. In sensitivity analysis 2, the OPLS-DA model was rebuilt using only the 18 endogenous VOCs with established biosynthetic routes, again with 1,000 permutations and leave-one-out cross-validation.

## 3 Results

### 3.1 Multivariate and univariate VOC profiling

After MetaboAnalyst preprocessing, 113 VOC features were retained. Univariate testing found 89 VOCs with FDR < 0.1 and 96 with |log2FC| > 0.26; 84 met both criteria. Principal component analysis (PCA) separated the CLP and sham cohorts along PC1 (Figure 1A). OPLS-DA with one predictive and one orthogonal component discriminated the groups (R2X = 0.489, R2Y = 0.981, Q2 = 0.777) (Figure 2A). The permutation test used the 1,000 label permutations stated in the Methods; none of the 1,000 permuted datasets reached the observed R2Y or Q2 (permuted R2Y 0.491–0.895; permuted Q2 −1.410–0.508), giving *P* = 0.001 for both. The submitted version reported *P* = 0.05, produced by the default permutation setting of the ropls function (20 permutations) rather than by the 1,000 permutations used here. A significant permutation result does not make the model a validated classifier, however. Refitting the model on 23 animals and classifying the withheld animal assigned 23 of 24 correctly (accuracy 95.8%; sensitivity 92.9%; specificity 100%; AUC 0.964), with one CLP animal falling on the sham side of the boundary. As no independent cohort was available to test the model, the supervised separation should be interpreted with caution.

**Figure 1:**
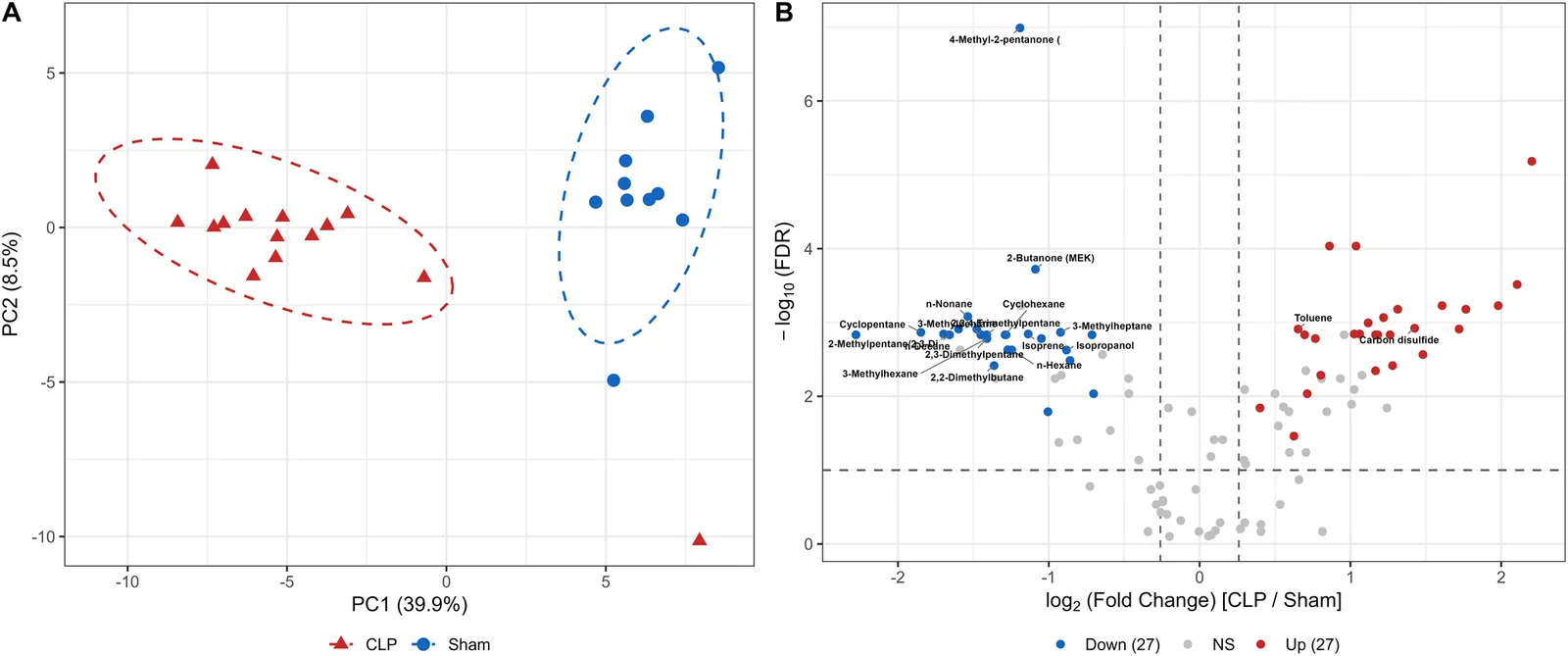
Multivariate and univariate profiling of systemic micro-environmental volatile organic compounds (VOCs). **(A)** Principal component analysis (PCA) score plot showing the unsupervised separation between the cecal ligation and puncture (CLP; red triangles, *n* = 14) and sham-operated control (blue circles, *n* = 10) groups. Ellipses indicate 95% confidence regions. **(B)** Volcano plot of −log10(FDR) versus log2(fold change, FC). Red and blue points represent VOCs significantly increased and decreased in the CLP cohort, respectively [screening criteria: FDR < 0.1, |log2FC| > 0.26, and variable importance in projection (VIP) > 1.0]; gray points are non-significant. Bold black labels highlight the 18 confirmed endogenous VOCs. VOC, volatile organic compound; CLP, cecal ligation and puncture; PCA, principal component analysis; FDR, false discovery rate; FC, fold change; VIP, variable importance in projection.

**Figure 2:**
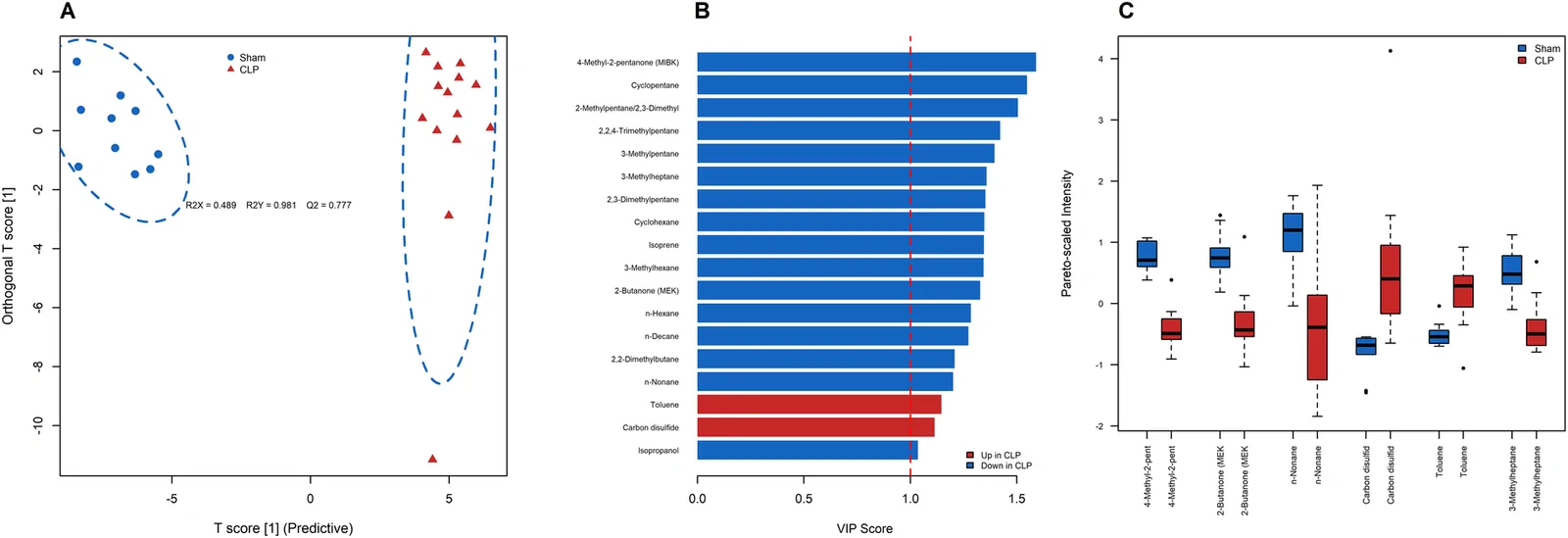
Supervised orthogonal partial least squares discriminant analysis (OPLS-DA) and expression profiles of endogenous VOCs. **(A)** OPLS-DA score plot (one predictive + one orthogonal component) showing inter-group discrimination with 95% confidence ellipses. Model parameters: R2X = 0.489 (fraction of X-variance explained), R2Y = 0.981 (fraction of Y-variance explained), Q2 = 0.777 (cross-validated predictive ability; 1,000-iteration permutation test: pR2Y = 0.001, pQ2 = 0.001). **(B)** VIP scores of the 18 identified endogenous VOCs. Red bars indicate increased abundance in the CLP group; blue bars indicate decreased abundance. The dashed red line represents the VIP = 1.0 threshold. **(C)** Box plots of the six most statistically significant endogenous VOCs ranked by FDR (Sham, blue; CLP, red). In each box plot, the horizontal line represents the median, hinges correspond to the first and third quartiles, and whiskers extend to the most extreme data point within 1.5 × the interquartile range; outliers are plotted individually. OPLS-DA, orthogonal partial least squares discriminant analysis; VIP, variable importance in projection; FDR, false discovery rate; R2X and R2Y, fractions of X- and Y-variance explained; Q2, cross-validated predictive ability; pR2Y and pQ2, permutation *P* values for R2Y and Q2; MIBK, 4-methyl-2-pentanone; MEK, 2-butanone; CLP, cecal ligation and puncture.

### 3.2 Candidate differential VOCs and endogenous classification

Applying FDR < 0.1, |log2FC| > 0.26, and VIP > 1.0 identified 54 candidate VOCs (Figure 1B). The top-ranked by FDR were 4-methyl-2-pentanone (MIBK; FDR = 1.0 × 10⁻⁷, log2FC = −1.19), dichloromethane (FDR = 6.6 × 10⁻⁶, log2FC =  + 2.20), and benzyl chloride (FDR = 5.9 × 10⁻⁴, log2FC =  + 1.61). Full statistics for all 54 candidates are in Supplementary Table S1. Several of the strongest discriminators were halogenated environmental compounds; their systematic elevation in the CLP cohort is taken up in the Discussion.

Literature curation split the 54 candidates into 18 (33%) endogenous VOCs with established mammalian or microbial origins, 16 (30%) endogenous VOCs without specific pathway mapping, and 20 (37%) exogenous compounds, mostly halogenated hydrocarbons and industrial solvents that also occur in ambient air. Sixteen of the 20 exogenous compounds were higher in the CLP cohort, so batch- or chamber-related background cannot be ruled out from the full model alone.

### 3.3 Metabolic pathway enrichment of endogenous VOCs

Hierarchical clustering of the 18 endogenous VOCs produced distinct expression signatures between the groups, with functionally related metabolites forming coherent clusters (Figure 3A). Pathway enrichment resolved four metabolic domains (Figure 3B).

**Figure 3:**
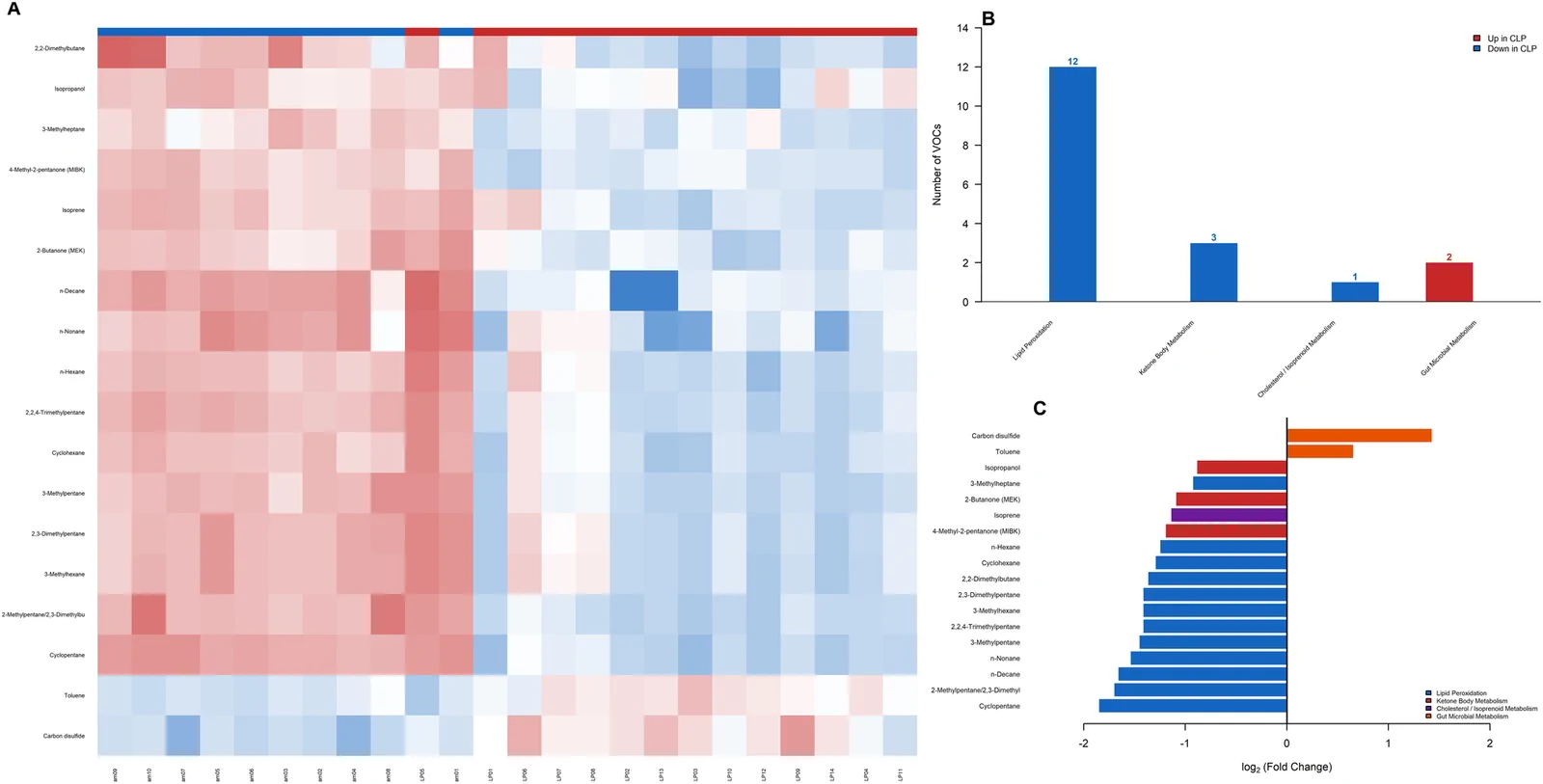
Metabolic pathway enrichment analysis of the endogenous volatilomic signature. **(A)** Hierarchical clustering heatmap of the 18 endogenous VOCs (Ward's minimum variance method, Euclidean distance). Each row represents a VOC and each column an individual animal (Sham01–Sham10, CLP01–CLP14). The colour scale represents Pareto-scaled abundance (red, increased; blue, decreased); the row sidebar denotes the assigned metabolic pathway category, and the column top bar denotes the experimental group (blue, Sham; red, CLP). **(B)** Pathway enrichment summary showing the number of VOCs significantly increased (red) or decreased (blue) within each metabolic category. **(C)** Waterfall plot of log2(fold change) for each endogenous VOC; bar colours correspond to the pathway categories in panel A. A positive log2FC indicates higher abundance in the CLP group relative to the sham group. VOC, volatile organic compound; log2FC, log2 fold change; CLP, cecal ligation and puncture.

The dominant signature came from lipid peroxidation and oxidative membrane damage, with 12 VOCs: linear alkanes (n-hexane, n-nonane, n-decane), branched alkanes (2-methylpentane/2,3-dimethylbutane, 2,2-dimethylbutane, 3-methylpentane, 2,3-dimethylpentane, 3-methylhexane, 2,2,4-trimethylpentane, 3-methylheptane), and cyclic alkanes (cyclopentane, cyclohexane). All 12 were depleted in the CLP group, with log2FC from −0.92 (3-methylheptane) to −1.85 (cyclopentane) (Figure 3C). The second domain covered ketone body metabolism: MIBK (log2FC = −1.19), 2-butanone (log2FC = −1.09), and isopropanol (log2FC = −0.88), all decreased. The third domain, cholesterol/isoprenoid metabolism, was represented by isoprene (log2FC = −1.14), classified separately because recent work links breath isoprene to skeletal-muscle IDI2 activity (31). The fourth domain was assigned to putative gut microbiota-associated metabolism, with toluene (log2FC =  + 0.65) and carbon disulfide (log2FC =  + 1.43) both elevated. Overall, 16 of the 18 endogenous VOCs fell in septic mice, and only these two putative microbiota-associated compounds rose. Pathway assignment, direction and proposed origin for all 18 compounds are listed in Supplementary Table S2.

### 3.4 Sensitivity analyses

The 18 endogenous VOCs and the four pathway domains survived both sensitivity analyses. Removing all 20 exogenous VOCs (Supplementary Table S3) left 93 features, and the univariate and multivariate structure was essentially unchanged: OPLS-DA on the restricted matrix gave R2X = 0.448, R2Y = 0.969, and Q2 = 0.716 (*P* = 0.001). Forty features met all three candidate criteria, and each of the 34 compounds that had been a candidate in the full analysis and was not exogenous remained a candidate. All 18 endogenous VOCs retained candidate status (worst FDR 0.0049; smallest VIP 1.08; smallest |log2FC| 0.65), every fold-change direction was identical to the full model, and the composition of the four pathway domains was unchanged (Supplementary Table S4). Rebuilding the model from the 18 endogenous VOCs alone also retained group separation (R2X = 0.826, R2Y = 0.860, Q2 = 0.774; *P* = 0.001; leave-one-out accuracy 95.8%, AUC 0.943). Because these compounds were selected using statistics from the full dataset, the second model is a robustness check rather than an independently validated classifier. Neither analysis supports the interpretation that the pathway-level pattern is carried by exogenous contaminants.

## 4 Discussion

The endogenous VOCs identified in septic mice form metabolically coherent categories rather than an arbitrary set of statistical hits. By restricting pathway enrichment to literature-verified endogenous metabolites, we mapped the septic volatilome onto four interconnected axes: suppressed lipid peroxidation, attenuated ketogenesis, reduced cholesterol/isoprenoid metabolism (isoprene), and putative gut microbiota-associated metabolism. The near-uniform depletion of host-derived VOCs (16 of 18) fits a generalised sepsis-associated hypometabolic state. Reduced physical activity and ventilation in septic mice could produce a very similar pattern, and the sections below are organized around that ambiguity.

### 4.1 Interpretation of the whole-body volatile profile and its limits

The VOCs analysed here were recovered from the headspace of a sealed chamber holding a whole animal, not from tissue or blood. What is measured is therefore the sum of superimposed contributions: emissions from host metabolism, the gut microbiota, skin and fur, residual ambient air, and the chamber itself. Three consequences follow. First, a compound's chamber concentration is not a direct readout of the activity of any single metabolic pathway. Second, because ventilation rate and physical activity both fall in septic mice, part of the uniform decline in host-derived volatiles could reflect reduced emission rather than reduced production; the present data cannot separate the two, and the same applies to isoprene, which is generated largely by skeletal muscle. Third, the design cannot attribute a compound to an organ; localizing a source would require exhaled-breath sampling from intubated animals, or *ex vivo* tissue headspace analysis with tissue metabolomics. The dataset therefore identifies which volatile features differ between CLP and sham animals and which link plausibly to known mammalian pathways; the mechanistic statements that follow are hypotheses generated from that pattern, not confirmations of *in vivo* pathway flux.

The uniform depletion of lipid peroxidation products is the largest single feature of the dataset. Straight-chain and branched alkanes in breath are classically produced by non-enzymatic oxidative degradation of polyunsaturated fatty acids (PUFAs) by reactive oxygen species (17, 22, 23). A decline in these products is compatible with reduced oxidative turnover of PUFA substrate, the interpretation developed in the next section. It is equally compatible with reduced pulmonary and cutaneous elimination, lower PUFA availability, or altered hepatic clearance. These possibilities are not mutually exclusive, and the data do not choose between them.

Sepsis-induced hypometabolism offers a second, compatible mechanism. Falls in cardiac output, systemic oxygen consumption, and thermoregulatory drive could lower reactive oxygen species generation and lipid peroxidation independently of substrate supply (19). Whole-body sampling adds a mechanical factor: septic mice move less and breathe less, which lowers the emission of activity-linked volatiles such as isoprene regardless of metabolic flux. The synchronized drop in all three ketone-related VOCs (MIBK, 2-butanone, isopropanol) points to suppressed hepatic fatty acid oxidation at 12 h, a pattern that fits the metabolic quiescence of immune and parenchymal cells during acute polymicrobial sepsis. Volatilomic data alone cannot separate impaired substrate delivery, mitochondrial dysfunction, and cytokine-mediated metabolic arrest.

The rise in toluene and carbon disulfide is compatible with sepsis-induced intestinal dysbiosis (25, 29); we describe these as putative gut microbiota-associated rather than gut-microbial-derived. Both appear regularly in the mammalian volatilome (29), have been tied to gut microbial aromatic amino acid and sulfur metabolism (25, 26), and fecal microbial metabolites also change in inflammatory states (20). Neither can be assigned a microbial origin from VOC data alone: both occur in ambient air, both were detectable at low levels in chamber blanks, and inflammation-induced suppression of hepatic clearance could also raise their concentrations. Confirming a microbial contribution would require antibiotic-mediated microbiota depletion, germ-free animals, or faecal microbiota transplantation under an identical sampling protocol. If real, this selective accumulation is consistent with gut barrier breakdown and increased translocation of microbial byproducts during sepsis (26).

Our data agree with earlier rodent breath studies on some points and diverge on others. Fink et al. found reduced short-chain ketones, specifically acetone and 3-pentanone, after cecal ligation and incision in rats (9); we likewise saw depletion of MIBK, 2-butanone, and isopropanol, consistent with suppressed ketogenesis, probably from cytokine-mediated inhibition of hepatic mitochondrial fatty acid *β*-oxidation (21). Guaman et al. reported 15 discriminatory targets in endotoxemia rats, five of which (alkanes and toluene) overlap with our molecular classes (10). The convergence across CLP, cecal incision, and lipopolysaccharide models, and across GC-MS and IMS platforms, suggests these volatile changes reflect shared metabolic features of severe systemic inflammation rather than artifacts of a single model.

Other explanations remain possible. Mechanically ventilated ARDS patients show lower alveolar alkane output that correlates with reduced cardiac output and impaired pulmonary gas exchange (24); similar circulatory or ventilatory changes may operate in our mice. Our reclassification of isoprene as a skeletal-muscle product of the mevalonate pathway fits this view: the fall in isoprene may mainly reflect reduced physical activity and muscle cholesterol metabolism, not lipid peroxidation (31).

Background contamination is a persistent problem in volatilomic work (9, 10), and standardisation remains an open challenge in clinical breath analysis (27). Both cohorts shared one specific pathogen-free facility with identical diet, bedding, and climate, and gas collection was randomized with respect to group. Annotation was conservative: only compounds with documented mammalian or microbial biosynthetic routes were classed as endogenous, and ambiguous entities were left out of pathway mapping. If impaired clearance, such as inflammation-induced suppression of hepatic cytochrome P450 enzymes (30), drove the signal, endogenous and exogenous compounds would both accumulate; instead, the depletion was selective and pathway-coherent. Two sensitivity analyses tested this directly: with all 20 exogenous VOCs removed, all 34 remaining candidates kept their status, all 18 endogenous VOCs still met every threshold and the pathway domains were unchanged, and rebuilding the model from the 18 endogenous VOCs alone retained group separation (Q2 = 0.774, *P* = 0.001). These results argue against the possibility that the pathway-level pattern is carried by halogenated contaminants, but they do not remove the underlying problem: quantitative blank subtraction was not performed, chamber blanks were used to classify compounds rather than to subtract them, and 16 of the 20 exogenous compounds were higher in the CLP cohort, so residual batch or chamber effects cannot be formally excluded. Had the core pathway patterns disappeared once environment-related compounds were removed, the metabolic inferences drawn here would require substantial revision. Future work should randomise batches explicitly, subtract blanks quantitatively, and include a field blank in every batch.

A separate question is whether these patterns distinguish polymicrobial sepsis from sterile acute stress. Suppressed volatile ketones and modified lipid degradation products also appear in sterile tissue trauma and hemorrhagic shock (9, 19), and Fink et al. showed that some markers are specific to infection while others signal acute cellular distress generally (9). We therefore treat the fingerprint as a composite phenotype: individual components may overlap with non-infectious systemic inflammatory response syndromes, but the combined host-microbial gaseous signature offers a distinctive window into severe polymicrobial infection.

### 4.2 Hypothesis generation: substrate competition between peroxidation and eicosanoid synthesis

One mechanism deserves labelling as a hypothesis rather than an interpretation. During acute sepsis, PUFAs such as arachidonic acid are diverted into the cyclooxygenase-2 (COX-2) and lipoxygenase (5-LOX) cascades to fuel pro-inflammatory eicosanoid synthesis (18). Sufficient diversion would deplete the substrate pool available for non-enzymatic peroxidation and lower alkane output, which is one way to account for the data. The idea is attractive because it ties the volatile signature to a well-described feature of sepsis immunometabolism and predicts that alkane depletion should track eicosanoid production.

It is also entirely untested. We did not measure COX-2 or 5-LOX expression or activity, eicosanoid concentrations, or tissue PUFA pools, and volatilomic profiling cannot supply any of these. Alternative explanations, including reduced reactive oxygen species generation secondary to hypometabolism and reduced ventilatory elimination of volatile products, fit the same observation. The hypothesis is presented to be tested, not accepted. Testing it would require measuring COX-2 and 5-LOX activity, eicosanoids, tissue PUFA composition, and volatile emission in the same animals, ideally with pharmacological or genetic interruption of the eicosanoid cascade.

### 4.3 Limitations and translational limitation

The study has several limits. First, the sample size (*n* = 24) is small: leave-one-out cross-validation classified 95.8% of animals correctly, but a single misclassification moves accuracy by more than four percentage points, and no independent cohort was analyzed, so that figure describes this dataset rather than expected performance. Second, annotations are MSI Level 2 (spectral library matches without authentic standards). Third, the design is single-time-point: sampling covered only 12 h after CLP, so the data are a static snapshot of a dynamic syndrome and cannot distinguish early from late metabolic change, or survivors from non-survivors. Fourth, 16 endogenous VOCs of unknown origin and 20 exogenous VOCs were excluded from pathway mapping; the former may hide uncharacterized metabolic routes, and the latter underline the problem of environmental background. Fifth, whole-body sampling was not normalized to respiratory rate, minute ventilation, or activity, and no clinical severity score or activity telemetry was recorded; in future work an inert tracer gas or a simultaneously measured CO_2_ concentration would help decouple metabolic emission from ventilatory clearance, and disease-severity scoring would allow the volatile profile to be related to injury severity. Sixth, this signature cannot separate infectious inflammation from sterile insults such as trauma or hemorrhagic shock; it is a composite phenotype of systemic acute stress rather than a fully infection-specific signal, since similar volatile changes follow sterile injury (9, 19). Seventh, the model parameters, while favorable, should not be read as diagnostic performance: R2Y and Q2 are high for a dataset of this size, and permutation testing does not correct for feature selection or replace an external test set.

## 5 Conclusion

This feasibility study shows that GC-MS-based whole-body volatilomics with literature-curated pathway enrichment can map VOC changes in experimental sepsis onto candidate metabolic pathways. The broad suppression of host-derived VOCs, dominated by depleted lipid peroxidation products and ketone bodies, together with elevated putative microbiota-associated volatiles, offers a coherent framework for sepsis-associated metabolic reprogramming. Several experimental dimensions follow from these limitations. Longitudinal profiling at 6, 12, and 24 h after CLP would separate acute metabolic change from progressive organ failure and relate the volatilome to survival. Including female and aged animals, adding a CLP arm treated with antibiotics, and adding sterile-injury controls would begin to isolate infection-specific features from generic acute-stress responses. Normalizing to an inert tracer or to CO_2_, and pairing volatile measurements with tissue metabolomics and eicosanoid profiling, would attach the volatile pattern to a mechanism rather than a correlation. While still pre-clinical, these findings support further development of volatile-based metabolic phenotyping in critical care.

## Data Availability

The raw data supporting the conclusions of this article will be made available by the authors, without undue reservation.
