# Supplementary material for: Whole-body volatile organic compound profiling reveals metabolic pathway perturbations in a murine polymicrobial sepsis model: a feasibility study
Source: Front Med (Lausanne). 2026 Oct 14;13:1978491. doi: 10.3389/fmed.2026.1978491 (PMC13713802; doi:10.3389/fmed.2026.1978491)
Supplement: Supplementary file 1 [file Table1.docx]

**Supplementary Material**

*Whole-body volatile organic compound profiling reveals metabolic pathway perturbations in a murine polymicrobial sepsis model: a feasibility study*

Feng WY, Chen Y, Zhang XY, Fu R, Hu Q, Mo FF*, Wang JF*

**Supplementary Table S1. Complete statistics of the 54 candidate differential VOCs.**

Candidate criteria: FDR < 0.1, |log2FC| > 0.26, and VIP > 1.0. mean_CLP / mean_Sham: mean Pareto-scaled intensity in each group.

| **VOC** | **log2FC** | **p-value** | **FDR** | **VIP** | **mean CLP** | **mean Sham** |
| --- | --- | --- | --- | --- | --- | --- |
| 4-Methyl-2-pentanone (MIBK) | -1.190 | 9.07e-10 | 1.02e-07 | 1.590 | -0.433 | 0.757 |
| Dichloromethane (DCM) | 2.203 | 1.16e-07 | 6.56e-06 | 1.514 | 0.830 | -1.373 |
| trans-1,3-Dichloropropene | 0.861 | 2.73e-06 | 9.23e-05 | 1.342 | 0.208 | -0.653 |
| 1,2,4-Trimethylbenzene.1 | 1.037 | 3.27e-06 | 9.23e-05 | 1.436 | 0.289 | -0.748 |
| 2-Butanone (MEK) | -1.088 | 8.40e-06 | 1.90e-04 | 1.327 | -0.312 | 0.777 |
| 1,1,2-Trichlorotrifluoroethane | 2.106 | 1.63e-05 | 3.06e-04 | 1.355 | 0.757 | -1.349 |
| Trichlorofluoromethane (CFC-11) | 1.980 | 4.35e-05 | 5.92e-04 | 1.283 | 0.737 | -1.243 |
| Benzyl chloride | 1.608 | 4.71e-05 | 5.92e-04 | 1.329 | 0.628 | -0.980 |
| 1,2-Dichlorotetrafluoroethane | 1.766 | 6.44e-05 | 6.62e-04 | 1.280 | 0.669 | -1.097 |
| 1,2-Dibromo-3-chloropropane | 1.314 | 6.05e-05 | 6.62e-04 | 1.586 | 0.494 | -0.820 |
| n-Nonane | -1.537 | 8.81e-05 | 8.30e-04 | 1.201 | -0.435 | 1.102 |
| Naphthalene | 1.219 | 9.87e-05 | 8.58e-04 | 1.524 | 0.331 | -0.888 |
| o-Xylene | 1.118 | 1.25e-04 | 1.01e-03 | 1.524 | 0.335 | -0.783 |
| Carbon disulfide | 1.426 | 1.59e-04 | 1.20e-03 | 1.114 | 0.615 | -0.811 |
| 1-Pentene/trans/cis-2-Pentene | -1.477 | 2.01e-04 | 1.23e-03 | 1.505 | -0.404 | 1.073 |
| 1,2-Pentadiene | -1.598 | 2.01e-04 | 1.23e-03 | 1.512 | -0.498 | 1.100 |
| Dibromomethane | 1.721 | 2.01e-04 | 1.23e-03 | 1.251 | 0.574 | -1.147 |
| Toluene | 0.653 | 2.06e-04 | 1.23e-03 | 1.146 | 0.156 | -0.497 |
| Cyclopentane | -1.848 | 2.53e-04 | 1.36e-03 | 1.548 | -0.607 | 1.241 |
| 3-Methylheptane | -0.922 | 2.53e-04 | 1.36e-03 | 1.357 | -0.384 | 0.538 |
| Isoprene | -1.137 | 3.17e-04 | 1.43e-03 | 1.346 | -0.291 | 0.845 |
| Vinyl chloride | 1.062 | 3.17e-04 | 1.43e-03 | 1.137 | 0.400 | -0.662 |
| 2-Methylpentane/2,3-Dimethylbutane | -1.697 | 3.17e-04 | 1.43e-03 | 1.505 | -0.563 | 1.134 |
| Isopropylbenzene (Cumene) | 1.025 | 3.17e-04 | 1.43e-03 | 1.463 | 0.400 | -0.626 |
| Methyl tert-butyl ether (MTBE) | -2.279 | 4.94e-04 | 1.47e-03 | 1.498 | -0.739 | 1.540 |
| 3-Methylpentane | -1.450 | 4.94e-04 | 1.47e-03 | 1.395 | -0.461 | 0.989 |
| Chloroprene | 0.697 | 4.94e-04 | 1.47e-03 | 1.028 | 0.331 | -0.365 |
| Tetrahydrofuran (THF) | -0.713 | 4.07e-04 | 1.47e-03 | 1.160 | -0.268 | 0.445 |
| Cyclohexane | -1.290 | 4.94e-04 | 1.47e-03 | 1.347 | -0.430 | 0.860 |
| Carbon tetrachloride | 1.181 | 4.94e-04 | 1.47e-03 | 1.456 | 0.320 | -0.861 |
| 2,2,4-Trimethylpentane | -1.411 | 4.94e-04 | 1.47e-03 | 1.421 | -0.390 | 1.021 |
| 1-Heptene | -1.447 | 4.94e-04 | 1.47e-03 | 1.409 | -0.413 | 1.034 |
| Methyl methacrylate | -1.284 | 4.94e-04 | 1.47e-03 | 1.425 | -0.485 | 0.799 |
| n-Decane | -1.658 | 4.94e-04 | 1.47e-03 | 1.272 | -0.450 | 1.208 |
| 1,3-Dichlorobenzene | 1.167 | 4.94e-04 | 1.47e-03 | 1.339 | 0.312 | -0.855 |
| 1,2-Dichlorobenzene | 1.263 | 4.94e-04 | 1.47e-03 | 1.372 | 0.355 | -0.908 |
| Acrylonitrile | -1.049 | 6.14e-04 | 1.65e-03 | 1.013 | -0.404 | 0.645 |
| 2,3-Dimethylpentane | -1.411 | 6.14e-04 | 1.65e-03 | 1.352 | -0.354 | 1.056 |
| 3-Methylhexane | -1.411 | 6.14e-04 | 1.65e-03 | 1.344 | -0.356 | 1.055 |
| cis-1,3-Dichloropropene.1 | 0.768 | 6.14e-04 | 1.65e-03 | 1.295 | 0.180 | -0.588 |
| Bromoform (Tribromomethane) | -1.272 | 8.91e-04 | 2.34e-03 | 1.009 | -0.419 | 0.853 |
| n-Hexane | -1.244 | 9.39e-04 | 2.36e-03 | 1.284 | -0.347 | 0.898 |
| Isopropanol | -0.883 | 9.62e-04 | 2.36e-03 | 1.035 | -0.207 | 0.675 |
| 1,1,1,2-Tetrachloroethane | 1.481 | 1.16e-03 | 2.72e-03 | 1.057 | 0.410 | -1.071 |
| 2-Chlorotoluene | -0.859 | 1.42e-03 | 3.27e-03 | 1.041 | -0.303 | 0.557 |
| 2,2-Dimethylbutane | -1.363 | 1.73e-03 | 3.84e-03 | 1.208 | -0.405 | 0.958 |
| Dibromochloromethane | 1.280 | 1.73e-03 | 3.84e-03 | 1.046 | 0.398 | -0.881 |
| Trichloroethylene | 1.166 | 2.11e-03 | 4.50e-03 | 1.035 | 0.306 | -0.860 |
| 1,3-Diethylbenzene | 0.804 | 2.57e-03 | 5.18e-03 | 1.139 | 0.190 | -0.614 |
| 1,4-Dioxane | 0.714 | 5.41e-03 | 9.23e-03 | 1.021 | 0.166 | -0.548 |
| m/p-Xylene | -0.703 | 5.47e-03 | 9.23e-03 | 1.010 | -0.260 | 0.443 |
| Isopropylbenzene (Cumene).1 | 0.401 | 9.17e-03 | 1.44e-02 | 1.013 | 0.169 | -0.232 |
| Chloroform (Trichloromethane) | -1.005 | 1.09e-02 | 1.62e-02 | 1.029 | -0.292 | 0.714 |
| 1,4-Diethylbenzene | 0.626 | 2.42e-02 | 3.46e-02 | 1.002 | 0.144 | -0.482 |

**Supplementary Table S2. The 18 endogenous VOCs after removal of the exogenous VOCs : fold change is compared with the full model, and candidate status in sensitivity analysis is shown by its FDR and VIP.**

None of the 18 compounds lost candidate status after the exogenous VOCs were removed, and every fold-change direction was preserved. The full-model VIP values differ slightly because VIP is derived from the OPLS-DA model, which contains 113 features here and 93 features in sensitivity analysis.

| VOC | Pathway | log2FC (full) | log2FC (SA1) | FDR (SA1) | VIP (SA1) |
| --- | --- | --- | --- | --- | --- |
| 4-Methyl-2-pentanone (MIBK) | Ketone body metabolism | -1.190 | -1.190 | 8.43e-08 | 1.707 |
| 2-Butanone (MEK) | Ketone body metabolism | -1.088 | -1.088 | 2.60e-04 | 1.413 |
| n-Nonane | Lipid peroxidation | -1.537 | -1.537 | 1.53e-03 | 1.300 |
| Carbon disulfide | Sulfur / gut microbiota | 1.426 | 1.426 | 1.74e-03 | 1.199 |
| Toluene | Aromatic amino acid / gut microbiota | 0.653 | 0.653 | 1.74e-03 | 1.213 |
| 3-Methylheptane | Lipid peroxidation | -0.922 | -0.922 | 1.81e-03 | 1.460 |
| Cyclopentane | Lipid peroxidation | -1.848 | -1.848 | 1.81e-03 | 1.658 |
| 2-Methylpentane/2,3-Dimethylbutane | Lipid peroxidation | -1.697 | -1.697 | 1.84e-03 | 1.612 |
| Isoprene | Cholesterol / isoprenoid | -1.137 | -1.137 | 1.84e-03 | 1.439 |
| Cyclohexane | Lipid peroxidation | -1.290 | -1.290 | 1.91e-03 | 1.454 |
| 3-Methylpentane | Lipid peroxidation | -1.450 | -1.450 | 1.91e-03 | 1.500 |
| 2,2,4-Trimethylpentane | Lipid peroxidation | -1.411 | -1.411 | 1.91e-03 | 1.526 |
| n-Decane | Lipid peroxidation | -1.658 | -1.658 | 1.91e-03 | 1.385 |
| 3-Methylhexane | Lipid peroxidation | -1.411 | -1.411 | 2.04e-03 | 1.451 |
| 2,3-Dimethylpentane | Lipid peroxidation | -1.411 | -1.411 | 2.04e-03 | 1.459 |
| n-Hexane | Lipid peroxidation | -1.244 | -1.244 | 2.89e-03 | 1.384 |
| Isopropanol | Ketone body metabolism | -0.883 | -0.883 | 2.89e-03 | 1.082 |
| 2,2-Dimethylbutane | Lipid peroxidation | -1.363 | -1.363 | 4.88e-03 | 1.284 |

**Supplementary Table S3. The 20 exogenous VOCs removed in sensitivity analysis, with their statistics in the full model.**

These compounds were assigned an exogenous origin by literature curation because they are halogenated hydrocarbons or industrial solvents that also occur in ambient air, and they were detected in the empty-chamber blanks collected alongside the biological samples. They are listed here so that the effect of their removal can be traced; their univariate statistics in the full model are given for reference.

| VOC | log2FC (full) | p-value (full) | FDR (full) | VIP (full) |
| --- | --- | --- | --- | --- |
| Dichloromethane (DCM) | 2.203 | 1.16e-07 | 6.56e-06 | 1.514 |
| trans-1,3-Dichloropropene | 0.861 | 2.73e-06 | 9.23e-05 | 1.342 |
| 1,1,2-Trichlorotrifluoroethane | 2.106 | 1.63e-05 | 3.06e-04 | 1.355 |
| Trichlorofluoromethane (CFC-11) | 1.980 | 4.35e-05 | 5.92e-04 | 1.283 |
| Benzyl chloride | 1.608 | 4.71e-05 | 5.92e-04 | 1.329 |
| 1,2-Dibromo-3-chloropropane | 1.314 | 6.05e-05 | 6.62e-04 | 1.586 |
| 1,2-Dichlorotetrafluoroethane | 1.766 | 6.44e-05 | 6.62e-04 | 1.280 |
| Dibromomethane | 1.721 | 2.01e-04 | 1.23e-03 | 1.251 |
| Vinyl chloride | 1.062 | 3.17e-04 | 1.43e-03 | 1.137 |
| Chloroprene | 0.697 | 4.94e-04 | 1.47e-03 | 1.028 |
| Carbon tetrachloride | 1.181 | 4.94e-04 | 1.47e-03 | 1.456 |
| Methyl tert-butyl ether (MTBE) | -2.279 | 4.94e-04 | 1.47e-03 | 1.498 |
| 1,2-Dichlorobenzene | 1.263 | 4.94e-04 | 1.47e-03 | 1.372 |
| 1,3-Dichlorobenzene | 1.167 | 4.94e-04 | 1.47e-03 | 1.339 |
| Bromoform (Tribromomethane) | -1.272 | 8.91e-04 | 2.34e-03 | 1.009 |
| 1,1,1,2-Tetrachloroethane | 1.481 | 1.16e-03 | 2.72e-03 | 1.057 |
| 2-Chlorotoluene | -0.859 | 1.42e-03 | 3.27e-03 | 1.041 |
| Dibromochloromethane | 1.280 | 1.73e-03 | 3.84e-03 | 1.046 |
| Trichloroethylene | 1.166 | 2.11e-03 | 4.50e-03 | 1.035 |
| Chloroform (Trichloromethane) | -1.005 | 1.09e-02 | 1.62e-02 | 1.029 |

**Supplementary Table S4. Univariate statistics and candidate status of the 93 features retained after removal of the 20 exogenous VOCs.**

Candidate criteria: FDR < 0.1, |log2FC| > 0.26, and VIP > 1.0, identical to the full analysis. log2FC is computed on Pareto-scaled intensities as described in the Methods. Rows are ordered as returned by the pipeline.

| VOC | log2FC | p-value | FDR | VIP | Candidate |
| --- | --- | --- | --- | --- | --- |
| Bromoethane | 1.242 | 9.17e-03 | 1.61e-02 | 1.047 | Yes |
| Isoprene | -1.137 | 3.17e-04 | 1.84e-03 | 1.439 | Yes |
| 1-Pentene/trans/cis-2-Pentene | -1.477 | 2.01e-04 | 1.74e-03 | 1.608 | Yes |
| Chloroethane | 0.593 | 1.09e-02 | 1.80e-02 | 0.608 | No |
| Carbon disulfide | 1.426 | 1.59e-04 | 1.74e-03 | 1.199 | Yes |
| n-Butane | -0.810 | 2.81e-02 | 4.22e-02 | 0.749 | No |
| 1,1-Dichloroethylene | 0.407 | 5.01e-01 | 5.54e-01 | 0.506 | No |
| Bromomethane | 0.843 | 1.09e-02 | 1.80e-02 | 0.810 | No |
| Acrylonitrile | -1.049 | 6.14e-04 | 2.04e-03 | 1.079 | Yes |
| Isopropanol | -0.883 | 9.62e-04 | 2.89e-03 | 1.082 | Yes |
| Isopentane/n-Pentane | -0.217 | 3.52e-01 | 4.09e-01 | 0.287 | No |
| 1,3-Butadiene | -0.342 | 6.61e-01 | 6.83e-01 | 0.345 | No |
| trans/cis-2-Butene | 0.078 | 7.42e-01 | 7.58e-01 | 0.171 | No |
| Acrolein | -0.244 | 2.28e-01 | 2.79e-01 | 0.378 | No |
| Acetone | -0.958 | 3.11e-03 | 6.88e-03 | 1.006 | Yes |
| trans-1,2-Dichloroethylene | -0.403 | 5.70e-02 | 7.80e-02 | 0.701 | No |
| 2,2-Dimethylbutane | -1.363 | 1.73e-03 | 4.88e-03 | 1.284 | Yes |
| 1,1-Dichloroethane | 0.299 | 4.64e-01 | 5.26e-01 | 0.455 | No |
| 1,2-Pentadiene | -1.598 | 2.01e-04 | 1.74e-03 | 1.621 | Yes |
| Cyclopentane | -1.848 | 2.53e-04 | 1.81e-03 | 1.658 | Yes |
| cis-1,2-Dichloroethylene | -0.243 | 2.16e-01 | 2.68e-01 | 0.612 | No |
| 2-Methylpentane/2,3-Dimethylbutane | -1.697 | 3.17e-04 | 1.84e-03 | 1.612 | Yes |
| Methyl acetate | -0.728 | 1.35e-01 | 1.75e-01 | 0.518 | No |
| 3-Methylpentane | -1.450 | 4.94e-04 | 1.91e-03 | 1.500 | Yes |
| n-Hexane | -1.244 | 9.39e-04 | 2.89e-03 | 1.384 | Yes |
| 2-Butanone (MEK) | -1.088 | 8.40e-06 | 2.60e-04 | 1.413 | Yes |
| 1-Hexene | -0.323 | 1.52e-01 | 1.91e-01 | 0.519 | No |
| cis-1,2-Dichloroethylene.1 | -0.199 | 7.92e-01 | 7.92e-01 | 0.085 | No |
| Ethyl acetate | 0.959 | 3.96e-04 | 1.91e-03 | 1.016 | Yes |
| Vinyl acetate | -0.004 | 6.61e-01 | 6.83e-01 | 0.050 | No |
| Tetrahydrofuran (THF) | -0.713 | 4.07e-04 | 1.91e-03 | 1.239 | Yes |
| Methylcyclopentane | 0.815 | 6.61e-01 | 6.83e-01 | 0.538 | No |
| 1,1,1-Trichloroethane | -0.125 | 4.29e-01 | 4.93e-01 | 0.161 | No |
| Cyclohexane | -1.290 | 4.94e-04 | 1.91e-03 | 1.454 | Yes |
| 2,4-Dimethylpentane | -1.185 | 4.71e-05 | 1.10e-03 | 0.882 | No |
| 1,1-Dichloropropene | 0.104 | 6.19e-01 | 6.69e-01 | 0.093 | No |
| Benzene | -0.288 | 2.54e-01 | 3.02e-01 | 0.297 | No |
| 1,2-Dichloropropane | 0.810 | 3.11e-03 | 6.88e-03 | 0.880 | No |
| 2,3-Dimethylpentane | -1.411 | 6.14e-04 | 2.04e-03 | 1.459 | Yes |
| 3-Methylhexane | -1.411 | 6.14e-04 | 2.04e-03 | 1.451 | Yes |
| 1,2-Dichloroethane | 0.706 | 4.30e-02 | 6.21e-02 | 0.915 | No |
| n-Heptane | -0.592 | 2.00e-02 | 3.21e-02 | 0.918 | No |
| Dichlorobromomethane | 0.407 | 6.61e-01 | 6.83e-01 | 0.291 | No |
| 2,2,4-Trimethylpentane | -1.411 | 4.94e-04 | 1.91e-03 | 1.526 | Yes |
| 1-Heptene | -1.447 | 4.94e-04 | 1.91e-03 | 1.512 | Yes |
| 1,4-Dioxane | 0.714 | 5.41e-03 | 1.06e-02 | 1.123 | Yes |
| Methylcyclohexane | -1.353 | 3.11e-03 | 6.88e-03 | 0.918 | No |
| Dichlorobromomethane.1 | -0.644 | 1.16e-03 | 3.36e-03 | 0.694 | No |
| Bromodichloromethane | -0.052 | 1.09e-02 | 1.80e-02 | 0.055 | No |
| Methyl methacrylate | -1.284 | 4.94e-04 | 1.91e-03 | 1.541 | Yes |
| 2,3,4-Trimethylpentane | 0.153 | 2.81e-02 | 4.22e-02 | 0.187 | No |
| 2-Methylheptane | 0.271 | 5.78e-01 | 6.32e-01 | 0.256 | No |
| 4-Methyl-2-pentanone (MIBK) | -1.190 | 9.07e-10 | 8.43e-08 | 1.707 | Yes |
| cis-1,3-Dichloropropene | 0.298 | 4.51e-03 | 9.54e-03 | 0.464 | No |
| cis-1,3-Dichloropropene.1 | 0.768 | 6.14e-04 | 2.04e-03 | 1.386 | Yes |
| 3-Methylheptane | -0.922 | 2.53e-04 | 1.81e-03 | 1.460 | Yes |
| trans-1,3-Dichloropropene.1 | 0.704 | 2.11e-03 | 5.78e-03 | 0.689 | No |
| Toluene | 0.653 | 2.06e-04 | 1.74e-03 | 1.213 | Yes |
| 1,1,2-Trichloroethane | -0.933 | 3.10e-02 | 4.58e-02 | 0.687 | No |
| n-Octane | 0.534 | 2.54e-01 | 3.02e-01 | 0.495 | No |
| 2-Hexanone | -0.263 | 1.30e-01 | 1.70e-01 | 0.599 | No |
| 1,3-Dichloropropane | 0.933 | 3.11e-03 | 6.88e-03 | 0.993 | No |
| Tetrachloroethylene (PCE) | 1.077 | 2.57e-03 | 6.45e-03 | 0.939 | No |
| Chlorodibromomethane | -0.471 | 2.90e-03 | 6.88e-03 | 1.090 | Yes |
| 1,2-Dibromoethane | 0.556 | 8.48e-03 | 1.58e-02 | 1.013 | Yes |
| Chlorobenzene | -0.469 | 5.41e-03 | 1.06e-02 | 0.902 | No |
| Ethylbenzene | -0.258 | 3.26e-01 | 3.84e-01 | 0.449 | No |
| m/p-Xylene | -0.703 | 5.47e-03 | 1.06e-02 | 1.085 | Yes |
| n-Nonane | -1.537 | 8.81e-05 | 1.53e-03 | 1.300 | Yes |
| Styrene | 0.293 | 5.63e-02 | 7.80e-02 | 0.864 | No |
| o-Xylene | 1.118 | 1.25e-04 | 1.67e-03 | 1.611 | Yes |
| Isopropylbenzene (Cumene) | 1.025 | 3.17e-04 | 1.84e-03 | 1.556 | Yes |
| 1,1,2,2-Tetrachloroethane | -0.917 | 2.57e-03 | 6.45e-03 | 0.897 | No |
| Isopropylbenzene (Cumene).1 | 0.401 | 9.17e-03 | 1.61e-02 | 1.050 | Yes |
| 1,2,3-Trichloropropane | 0.596 | 4.34e-02 | 6.21e-02 | 0.979 | No |
| n-Propylbenzene | 0.522 | 1.71e-02 | 2.80e-02 | 0.923 | No |
| 4-Chlorotoluene | 0.306 | 6.51e-02 | 8.78e-02 | 0.222 | No |
| 2-Ethyltoluene | 0.137 | 4.69e-01 | 5.26e-01 | 0.156 | No |
| 1,3,5-Trimethylbenzene.1 | 0.059 | 7.74e-01 | 7.83e-01 | 0.014 | No |
| 1,2,4-Trimethylbenzene | -0.025 | 1.51e-01 | 1.91e-01 | 0.038 | No |
| 1,2,4-Trimethylbenzene.1 | 1.037 | 3.27e-06 | 1.52e-04 | 1.507 | Yes |
| n-Decane | -1.658 | 4.94e-04 | 1.91e-03 | 1.385 | Yes |
| tert-Butylbenzene | 1.008 | 7.72e-03 | 1.46e-02 | 0.866 | No |
| sec-Butylbenzene | 0.096 | 2.81e-02 | 4.22e-02 | 0.104 | No |
| 1,2,3-Trimethylbenzene | 0.500 | 5.41e-03 | 1.06e-02 | 0.724 | No |
| 4-Isopropyltoluene (p-Cymene) | 1.025 | 4.51e-03 | 9.54e-03 | 0.782 | No |
| n-Butylbenzene | -0.207 | 9.17e-03 | 1.61e-02 | 0.235 | No |
| 1,3-Diethylbenzene | 0.804 | 2.57e-03 | 6.45e-03 | 1.219 | Yes |
| 1,4-Diethylbenzene | 0.626 | 2.42e-02 | 3.81e-02 | 1.068 | Yes |
| n-Undecane | -1.587 | 9.39e-04 | 2.89e-03 | 1.034 | Yes |
| Naphthalene | 1.219 | 9.87e-05 | 1.53e-03 | 1.630 | Yes |
| n-Dodecane | 0.075 | 4.98e-02 | 7.02e-02 | 0.018 | No |
| Hexachloro-1,3-butadiene | 0.659 | 1.07e-01 | 1.43e-01 | 0.545 | No |
